# Cerebrovascular Reactivity Mapping Without Gas Challenges: A Methodological Guide

**DOI:** 10.3389/fphys.2020.608475

**Published:** 2021-01-18

**Authors:** Joana Pinto, Molly G. Bright, Daniel P. Bulte, Patrícia Figueiredo

**Affiliations:** ^1^Institute of Biomedical Engineering, Department of Engineering Science, University of Oxford, Oxford, United Kingdom; ^2^Institute for Systems and Robotics - Lisboa and Department of Bioengineering, Instituto Superior Técnico, Universidade de Lisboa, Lisbon, Portugal; ^3^Physical Therapy and Human Movement Sciences, Feinberg School of Medicine, Northwestern University, Chicago, IL, United States; ^4^Biomedical Engineering, McCormick School of Engineering, Northwestern University, Evanston, IL, United States

**Keywords:** cerebrovascular reactivity, MRI, breath-hold, paced deep breathing, resting-state

## Abstract

Cerebrovascular reactivity (CVR) is defined as the ability of vessels to alter their caliber in response to vasoactive factors, by means of dilating or constricting, in order to increase or decrease regional cerebral blood flow (CBF). Importantly, CVR may provide a sensitive biomarker for pathologies where vasculature is compromised. Furthermore, the spatiotemporal dynamics of CVR observed in healthy subjects, reflecting regional differences in cerebral vascular tone and response, may also be important in functional MRI studies based on neurovascular coupling mechanisms. Assessment of CVR is usually based on the use of a vasoactive stimulus combined with a CBF measurement technique. Although transcranial Doppler ultrasound has been frequently used to obtain global flow velocity measurements, MRI techniques are being increasingly employed for obtaining CBF maps. For the vasoactive stimulus, vasodilatory hypercapnia is usually induced through the manipulation of respiratory gases, including the inhalation of increased concentrations of carbon dioxide. However, most of these methods require an additional apparatus and complex setups, which not only may not be well-tolerated by some populations but are also not widely available. For these reasons, strategies based on voluntary breathing fluctuations without the need for external gas challenges have been proposed. These include the task-based methodologies of breath holding and paced deep breathing, as well as a new generation of methods based on spontaneous breathing fluctuations during resting-state. Despite the multitude of alternatives to gas challenges, existing literature lacks definitive conclusions regarding the best practices for the vasoactive modulation and associated analysis protocols. In this work, we perform an extensive review of CVR mapping techniques based on MRI and CO_2_ variations without gas challenges, focusing on the methodological aspects of the breathing protocols and corresponding data analysis. Finally, we outline a set of practical guidelines based on generally accepted practices and available data, extending previous reports and encouraging the wider application of CVR mapping methodologies in both clinical and academic MRI settings.

## Introduction

Cerebrovascular reactivity (CVR) is an intrinsic regulatory mechanism whereby cerebral blood vessels adjust their caliber in response to a vasoactive stimulus, increasing or decreasing the regional cerebral blood flow (CBF). Importantly, CVR is thought to be a sensitive biomarker of the brain’s vascular health in a wide range of conditions and pathologies, including stroke (Krainik et al., 2005; Geranmayeh et al., 2015), glioma (Hsu et al., 2004; Pillai and Zacá, 2011; Zacà et al., 2014; Iranmahboob et al., 2016), small vessel disease (Hund-Georgiadis et al., 2003; Conijn et al., 2012), and obstructive sleep apnea (Prilipko et al., 2014; Buterbaugh et al., 2015; Ponsaing et al., 2018; Wu et al., 2019). Even in the healthy brain, CVR exhibits spatiotemporal patterns possibly reflecting regional variations in cerebral vasculature (Chang et al., 2008; Bright et al., 2009; Pinto et al., 2016), which may impact commonly used hemodynamic measures of brain activity, namely blood oxygen level dependent (BOLD) functional MRI (fMRI) (Ogawa et al., 1990). To take this into account, CVR mapping has been used as a normalizing factor in fMRI studies in order to reduce inter- and intrasubject variability (Handwerker et al., 2007; Thomason et al., 2007; Tsvetanov et al., 2015).

In order to evaluate CVR, a challenge to the vasculature is usually applied while the associated CBF changes are measured. While Transcranial Doppler Ultrasound (TCD) allows only global and indirect CVR evaluation, Positron Emission Tomography (PET) and Single-Photon Emission Computed Tomography (SPECT) enable whole brain CVR mapping; however, these techniques involve the administration of radiotracers, which can limit their ease of use and appropriateness in many studies. By overcoming both limitations, and further allowing improved spatial and temporal resolution, MRI techniques have become increasingly popular for CVR assessment. The BOLD contrast has been particularly useful in this context, as a surrogate of relative CBF changes. Although the BOLD signal results from a complex combination of several physiological parameters, including not only CBF but also cerebral blood volume (CBV) and blood oxygenation, it is nevertheless thought to reflect predominantly CBF changes (Mandell et al., 2008; Chen and Pike, 2010). For truly quantitative measurements of CBF, non-invasive Arterial Spin Labeling (ASL) perfusion imaging can be used (Detre et al., 1992; Williams et al., 1992; Alsop et al., 2015). However, the low signal-to-noise ratio (SNR) inherent to this type of measurement has limited its use. CVR values obtained with MRI methodologies have been shown to correlate with the ones obtained with gold standard techniques for CBF quantification (PET, SPECT) (Heijtel et al., 2014; Fierstra et al., 2018; Hauser et al., 2019).

Regarding the vascular challenge, the most common approach involves the induction of hypercapnia, whereby the arterial blood partial pressure of carbon dioxide (CO_2_) is increased leading to vasodilation and increased CBF. These vascular tone changes are thought to be mainly induced by CO_2_-mediated changes in pH levels (Kontos et al., 1977; Brian, 1998). A relatively common method of inducing hypercapnia is the inhalation of air with an increased CO_2_ partial pressure. For this purpose, a gas mixture with an altered concentration of CO_2_ (most commonly ∼5%) in relation to normal “room” air (consisting of approximately 21% O_2_ and 0.04% CO_2_ with balance nitrogen) is generally used. Different techniques have been developed and improved along the years in order to more precisely control gas concentrations. In particular, some respiratory gas manipulation techniques allow precise targeting of end-tidal carbon dioxide (PETCO_2_) and/or oxygen (PETO_2_) concentrations, which provide surrogates for the corresponding arterial gas concentrations (Mark et al., 2010; Spano et al., 2013). Nevertheless, the required experimental setups are quite complex, and for this reason may be associated with low tolerance by some clinical populations or be inappropriate in hospital scanning environments. Moreover, the required setups are quite expensive and may therefore simply not be available in many MRI facilities. Other less expensive gas delivery setups might also be used (Tancredi et al., 2014; Liu et al., 2017b), but these still require additional time for setting up the patient as well as expertise and equipment for manipulating gas levels.

Alternatively, strategies based on breathing tasks performed by the subjects without the need for external gas manipulations have been proposed: breath holding or paced deep breathing can readily induce changes in arterial pressure of blood gases and consequently drive vasodilation and vasoconstriction, respectively. Furthermore, a new generation of methods that are simply based on the spontaneous breathing fluctuations occurring during resting-state has also started to emerge. Both strategies have been shown to be well tolerated by populations generally considered less cooperative (Thomason et al., 2005; Handwerker et al., 2007; Kannurpatti et al., 2010; Raut et al., 2016; Dlamini et al., 2018) and to yield results comparable to those obtained with external gas manipulation techniques (Kastrup et al., 2001; Kannurpatti and Biswal, 2008; Golestani et al., 2016b; Liu et al., 2017a). Because of their simplicity and inexpensive implementation, these strategies might offer a new opportunity for the application of CVR mapping as a useful clinical and research tool.

The aim of this work is to review techniques for assessing CVR during MRI that do not require external gas manipulation. For this purpose, we will focus on the BOLD contrast for the MRI assessment of relative CBF changes, and we will consider the different methods that can be used to elicit a vascular response without the need for external gas manipulations. These include *task-based methods*, which rely on the voluntary execution of breathing tasks; and *resting-state methods*, which rely simply on the intrinsic breathing variations during an undirected or “resting” state. In each case, we first describe the experimental design and data acquisition for the respective vasoactive modulation approach, and we then discuss data analysis and modeling methodologies to derive CVR maps. A few reviews have addressed related aspects of CVR mapping: a summary of some of the stimuli commonly used to evaluate CVR, with the exception of resting-state methods (Fierstra et al., 2013; Moreton et al., 2016); technical reviews of CVR mapping, mainly focusing on CO_2_ inhalation methods (Moreton et al., 2016; Liu et al., 2019); and a systematic review of BH-based CVR mapping (Urback et al., 2017). Nevertheless, our review differs from these previous works by incorporating CVR methods based on spontaneous breathing fluctuations during resting-state and comparing them with task-based methodologies, and in this way giving a more comprehensive overview of the different methodologies for CVR assessment that do not require gas challenges. Additionally, we propose a set of practical guidelines for CVR mapping, addressing both acquisition and analysis methodologies, based on generally accepted practices and available data as described in this review.

## Task-Based Methods

**FIGURE 1:**
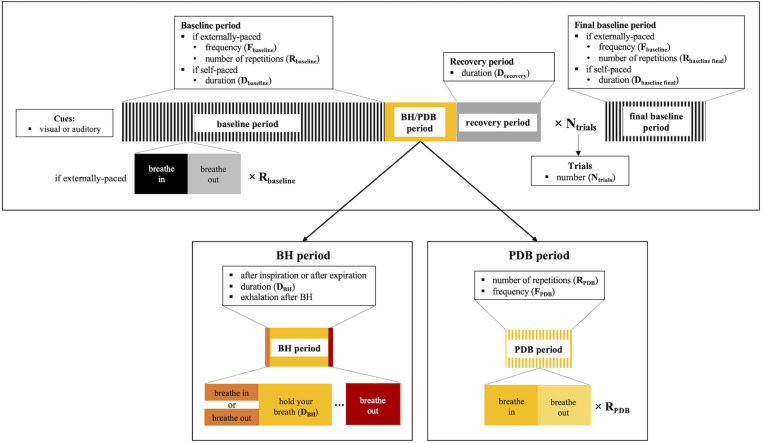
Schematic illustration of the breathing task paradigms (top), including details for breath-hold (BH, **bottom left**) and paced deep breathing (PDB, **bottom right**). The parameters and corresponding options that need to be considered for applying the breathing tasks are summarized.

This category of methods encompasses the voluntary execution of breathing tasks, designed to induce changes in the arterial pressure of CO_2_. These include hypercapnia-inducing breath-holding (BH), as well as hypocapnia-inducing paced deep breathing (PDB) tasks (Table 1). In both tasks, several parameters and options need to be considered before implementation. We summarize these in Figure 1, depicting the design of each task. A more detailed description will be provided in the following subsections.

**TABLE 1:** Summary of the most common non-invasive methodologies to induce a vascular response, their impact on arterial CO_2_ levels, cerebral vessels caliber and BOLD signal.

Task	PETCO_2_ levels	Vessel caliber	BOLD contrast
BH	↑	vasodilation	↑
PDB	↓	vasoconstriction	↓

### The Breath-Hold Task

The use of the BH task for CVR measurement was first described by Ratnatunga and Adiseshiah (1990), and it has since been the most common voluntary challenge chosen to elicit a cerebrovascular response. This task increases arterial CO_2_ levels due to subjects holding their breath and ceasing ventilation, ultimately leading to dilation of vasculature and an increase in CBF. The BH task offers a readily accessible and simple method for voluntary breathing modulation being reproducible at 1.5 and 3T (Magon et al., 2009; Lipp et al., 2015; Pinto et al., 2016; Peng et al., 2020).

Despite being used mostly in studies of healthy subjects, their successful application in children and elderly healthy populations indicates that BH tasks can be used to measure CVR even in populations that are generally considered less cooperative (Riecker et al., 2003; Thomason et al., 2005; Handwerker et al., 2007; Kannurpatti et al., 2010; Thomas et al., 2013; Raut et al., 2016). In fact, BH tasks have also been successfully applied in patients with pathologies, such as stroke (Raut et al., 2016), obstructive sleep apnea (Prilipko et al., 2014; Buterbaugh et al., 2015; Ponsaing et al., 2018), schizophrenia (Friedman et al., 2008) and glioma (Hsu et al., 2004; Iranmahboob et al., 2016). Other works have investigated the relationship between BH-CVR measurements and factors such as vascular risk/hypertension (Haight et al., 2015; Tchistiakova et al., 2015), smoking (Friedman et al., 2008), altitude/diving (Yan et al., 2011; Vestergaard and Larsson, 2019) and physical activity (Gonzales et al., 2014; Svaldi et al., 2015, 2017). Furthermore, some studies have reported high correlations between CVR mapping obtained using BH compared with methods based on CO_2_ inhalation (Kastrup et al., 2001; Biswal et al., 2007; Kannurpatti and Biswal, 2008; Tancredi and Hoge, 2013).

A BH task typically consists of a protocol that follows a standard block design, with alternating periods of breath holding and normal breathing. Nevertheless, there are several parameters and options, shown schematically in Figure 1, that need to be considered when implementing such a task.

#### BH Period Duration

When selecting the BH period duration, two primary factors must be balanced: the BH must be short enough to be successfully performed by the participant but long enough to induce a robust hypercapnia and CVR response (Kastrup et al., 1999b; Andrade et al., 2006; Magon et al., 2009). The CBF response to CO_2_ increases with the BH duration, up to certain limit, leading to an increase in the measured imaging signal amplitude and yielding higher sensitivity and SNR as well as more reproducible results (Magon et al., 2009). Nevertheless, shorter BH durations are easier to tolerate, particularly in clinical settings (van Oers et al., 2018), and may also be less susceptible to head motion artifacts. In fact, longer BH periods may be more challenging for the participant, which can result in large “recovery” breaths upon completion of the BH introducing severe head motion confounds (Thomason et al., 2007; Bright et al., 2009). Whether a BH is performed before an inspiration or an expiration highly influences the level of tolerability of the BH duration (discussed below).

Significant hypercapnia states have been reported with BH periods as short as 6 s (Abbott et al., 2005). Most studies adopt a feasible BH duration between 10 and 30 s, although the spatial extent of the significant BOLD response reaches a plateau at a BH length of approximately 20 s (Liu et al., 2002). Several studies have successfully applied BH with a duration of ∼15 s, hence this value might be a good compromise for BH CVR assessment (Leoni et al., 2008; Chang et al., 2009; Pattinson et al., 2009; Bright et al., 2011; Hua et al., 2011; Lipp et al., 2014, 2015; Tchistiakova et al., 2014; Geranmayeh et al., 2015; Urback et al., 2019, 2018).

#### End-Expiration or End-Inspiration BH

**FIGURE 2:**
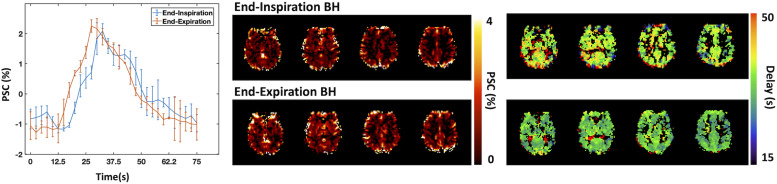
Comparison between end-inspiration (blue) and end-expiration (red) BH protocols: BOLD percent signal change (PSC) time-courses averaged across BH trials and across brain regions displaying significant signal changes. Error bars represent standard deviation across trials. Corresponding CVR maps representing the amplitude **(top)** and delay **(bottom)** of the BOLD response (due to the lack of PETCO_2_ information, the CVR amplitude values are presented in non-normalized units of absolute PSC). Maps were obtained using a Fourier basis modeling approach (Pinto et al., 2016).

End-inspiration BH yields more complex BOLD responses than end-expiration protocols, commonly exhibiting a triphasic shape, with an initial short positive phase, then a signal decrease followed by a major increase that reaches a maximum, and finally returning slowly back to baseline (Kastrup et al., 1998; Li et al., 1999a; Magon et al., 2009) (Figure 2). End-expiration BH displays a simpler response, without the first signal increase commonly observed in end-inspiration BH strategies. End-expiration BH protocols also tend to achieve faster responses than end-inspiration BH protocols. Nevertheless, if these timing differences are adequately taken into account, both BH protocols have been shown to yield comparable CVR amplitude results (Kastrup et al., 1999b) (Figure 2).

End-inspiration BH tasks are naturally easier to perform, allowing for longer BH period durations, and may therefore be more suitable for potentially less cooperative patients (Roberts et al., 2009; Pillai and Mikulis, 2014). Nevertheless, when using an end-inspiration BH task, the amplitude of the BOLD response also depends on the depth of the preparatory inspiration, although visual feedback during task inspiration period has been proposed to tackle this issue (Thomason and Glover, 2008).

End-expiration tasks have been shown to be reproducible (Scouten and Schwarzbauer, 2008) but may induce additional head motion due to the urge to inspire toward the end of the task (Thomason et al., 2007). Nevertheless, chest position during the BH is more similar across trials when following exhalation rather than an arbitrary inhalation, with more consistent field changes due to chest position. Alternatively, instead of employing an explicit end-inspiration or end-expiration BH protocol, some studies instruct subjects to cease breathing wherever they are in their natural breathing cycle (Bright et al., 2009, 2011).

#### Baseline and Recovery Periods

Regarding the baseline periods between BH, these usually have a long duration in order to allow blood gas levels to return to baseline (Poulin et al., 1996). Another aspect that may be modulated is the breathing rhythm during these periods, which may be spontaneous (self-paced) or paced (externally, computer-paced). The latter may help minimize variability inherent to the spontaneous breathing rates within and between subjects, resulting in a more consistent baseline condition (Scouten and Schwarzbauer, 2008). On the other hand, computer-paced breathing methods require the choice of an appropriate breathing frequency for achieving a normocapnic baseline state, and this is highly dependent on physiological variations in lung function and breathing depth, within and across subjects. Breathing rates from 1/3 Hz to 1/6 Hz have been reported in literature (Handwerker et al., 2007; Chang et al., 2008; Scouten and Schwarzbauer, 2008; Bright and Murphy, 2013; Lipp et al., 2014, 2015; Wu et al., 2015; Cohen and Wang, 2019). However, Bright and Murphy observed mild hypocapnia when using 1/6 Hz compared to self-paced acquisition (Bright and Murphy, 2013). Using a higher breathing rate (1/3.75 Hz), Tancredi and Hoge also observed lower CO_2_ values in comparison to self-paced baseline values (Tancredi and Hoge, 2013). Residual effects after controlling for breathing rate may be related to the fact that breathing may automatically become deeper when paced, which could also lead to hypocapnia (Bright and Murphy, 2013). Targeting the breathing frequency to match the participant’s spontaneous breathing rate might minimize these hypocapnic effects of computer-paced breathing.

If a paced breathing approach is used for the baseline period, an additional recovery period, consisting of several seconds of spontaneous breathing, is commonly introduced after the BH and exhalation period, before resuming the baseline period (Bright and Murphy, 2013; Cohen and Wang, 2019). This recovery period primarily acts to accommodate faster and deeper breaths, as needed, following completion of the BH, which may minimize participant discomfort or anxiety and increase compliance when returning to the paced breathing pattern.

#### Number of BH Trials

In principle, the more BH trials are executed within a scan session the more averaging can be done and thus the higher measurement signal-to-noise ratio can be achieved. However, more trials also lead to longer protocol durations, which may increase motion artifacts and patient fatigue or non-compliance. Lipp and colleagues assessed the reproducibility of CVR measurements using different numbers of trials, with the recommendation of at least 3 to guarantee reproducible maps, when a breath-hold duration of 15 s is used in combination with 18 s of paced breathing (Lipp et al., 2015).

### The Paced Deep Breathing Task

Despite the BH task being a valuable method to induce hypercapnia and vasodilation non-invasively, techniques to induce hypocapnia and vasoconstriction can also be useful, in particular when further vasodilation is compromised (Zhao et al., 2009; Bright et al., 2011). In that case, hyperventilation might provide a valuable alternative breathing task, as an increase in respiration rate/depth induces hypocapnia. Numerous studies have used this approach for CVR assessment in both healthy and patient populations (Posse et al., 1997; Weckesser et al., 1999; Cohen et al., 2002; Naganawa et al., 2002; Hund-Georgiadis et al., 2003; Krainik et al., 2005; Zhao et al., 2009; Hajjar et al., 2010; Tancredi and Hoge, 2013). However, prolonged hyperventilation periods may cause undesired effects such as light-headedness, dizziness, visual disturbance, numbness and paresthesia, palpitations, tachycardia, shakiness, tension or anxiety, panic attacks, and weakness or exhaustion (Posse et al., 1997). Moreover, hyperventilation tasks are usually associated with high levels of head motion synchronized with the inspiration/expiration cycle.

**FIGURE 3:**
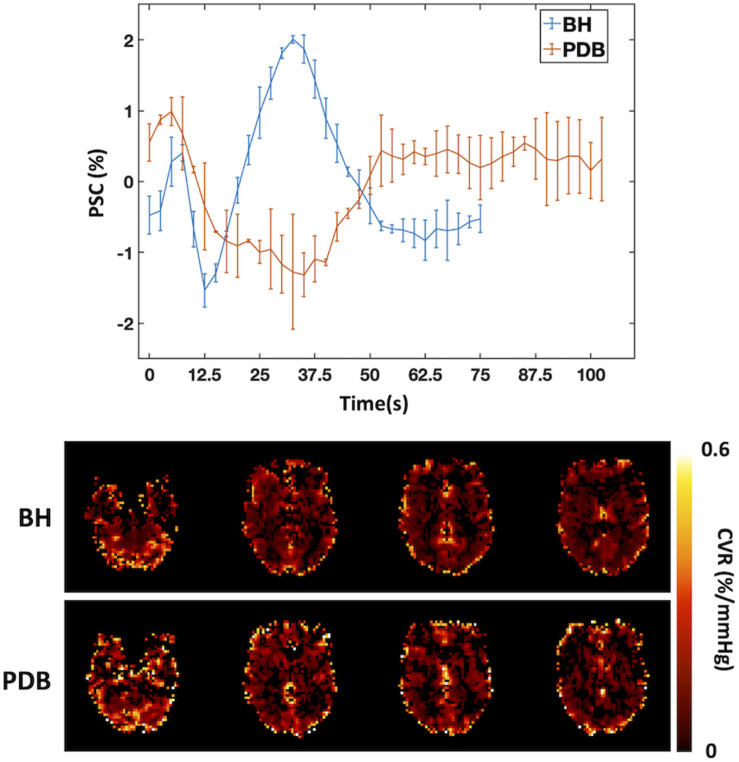
Comparison between BH and PDB protocols: Example of BOLD signal time courses averaged across trials and across regions that display significant signal responses (**left**, error bars represent standard deviation across trials), and corresponding CVR maps (in normalized units of BOLD percent signal change per mmHg (%/mmHg), **right**), for one illustrative subject. The BOLD signal changes in response to the different tasks can be seen clearly, with a signal increase/decrease for BH/PDB, respectively. A Fourier basis modeling approach was used to obtain the CVR maps (Pinto et al., 2016).

In order to overcome such limitations, paced breathing tasks have been designed to induce mild and transient hypocapnia. Bright et al. (2009) introduced a PDB paradigm consisting in a controlled and mild increase of respiration rate for brief periods of time. This method was compared with more standard approaches, such as BH or CO_2_ inhalation, yielding consistent results (Bright et al., 2009; Figure 3). In another study, Sousa et al. showed that a similar PDB protocol yielded CVR measurements with good within- and between-subject reproducibility (Sousa et al., 2014). Additionally, Bright et al. compared PDB and BH tasks during CO_2_ inhalation, mimicking cases where further basal vasodilation may be limited, and observed that PDB-derived CVR values (but not BH-derived CVR values) were significantly increased during this basal change in vascular tone (Bright et al., 2011). It is worth mentioning that a linear relationship between CBF and PETCO_2_ is assumed in the interpretation of these results, but this would not hold for extreme deviations of CO_2_ levels (more details in the section Other Considerations).

The PDB protocol typically follows a standard block design, with alternating periods of paced deep breathing and normal breathing. However, some parameters and options (shown schematically in Figure 1) need to be selected before implementing a PDB task.

#### Frequency, Depth and Duration of PDB

While some studies instruct subjects to hyperventilate without imposing a fixed rate or depth of respiration, others opt to specify this. In some studies, subjects practice the PDB task outside the scanner while PETCO_2_ levels are monitored: this can be done simply in order to verify their compliance (Posse et al., 1997; Zhao et al., 2009), or also to determine the subject-specific rate that best yields the required hypocapnic effect to be used in the final protocol (Cohen et al., 2002). Respiration rates of 1/4, 1/5 and 1/2.5 Hz have been previously used (Bright et al., 2009; Vogt et al., 2011; Sousa et al., 2014) in blocks of only a few breaths (2 or 3 breaths) (Bright et al., 2009, 2011) or for longer periods (8 breaths) (Sousa et al., 2014). Vogt and colleagues studied the impact of faster (1/2.5 Hz) and deeper (at a different frequency, 1/5 Hz) paced respiration for 20 s periods. As expected, increasing the breathing rate and depth decreased PETCO_2_ levels, but with faster breathing achieving lower values. Nevertheless, the combination of fast and deep breaths yields the lowest PETCO_2_ values compared to either fast or deep breathing separately (Vogt et al., 2011).

#### Baseline and Recovery Period

As in the case of BH tasks, PDB periods are alternated with “normal” breathing periods that act as a reference baseline. The duration of these baseline periods has varied between 60 and 90 s (Bright et al., 2009, 2011; Sousa et al., 2014). Although these studies employed self-paced breathing, Vogt et al. (2011) observed that PETCO_2_ decreases were greater when using external, computer-paced breathing compared to self-paced breathing (Vogt et al., 2011).

Deep recovery breaths are not typically required after performing PDB, but it may be more comfortable or natural for the participant to take slower or more shallow breaths following an hypocapnic task. It may therefore aid in improving participant comfort and compliance to include a short recovery period prior to the resumption of a paced baseline breathing pattern.

#### Number of Trials

The number of PDB trials has been highly dependent on the duration of each PDB period. While Sousa et al. used longer PDB periods and only two trials (Sousa et al., 2014), Bright et al. used shorter PDB periods but six trials (Bright et al., 2009). At present, there has not been a systematic assessment of the number of trials required for reliable CVR mapping using PDB.

### Additional Practices

#### Monitoring

Monitoring of task performance through the measurement of complementary physiological variables is strongly recommended, particularly PETCO_2_ levels. This can be achieved by using a capnograph in combination with a nasal cannula to measure the CO_2_ levels expired air. CO_2_ sampling and measurement should ideally be performed as close to the subject’s expired air flow (nostrils) as possible, as reducing the sample tube length diminishes dispersion effects as well as the time delay between the true physiological effect and measured recording (Moreton et al., 2016). However, the logistics of the MRI environment typically require several meters of tubing between the nasal cannula, near the center of the scanner bore, and the capnograph, which must be located in the control room. The sampling delay caused by this physical distance is impacted by the power of the capnograph’s vacuum pump, and a high vacuum strength is recommended. One simple way to determine the effective sampling delay is to record the CO_2_ trace following a single BH and measure the time between the end of the BH and the first end-tidal CO_2_ measurement after the BH (Bulte and Wartolowska, 2017).

The PETCO_2_ levels are extracted by identifying the maximum peaks on the recorded CO_2_ trace, and the final PETCO_2_ time course is obtained by correcting for the sampling delay and interpolating to the time of each acquired fMRI volume (Bulte and Wartolowska, 2017). It is worth noting that, during a BH, no information is collected, and therefore the most important PETCO_2_ measurements in each trial are from the exhalations immediately before and after the BH period. It is important to emphasize that these exhalations are crucial for obtaining the most accurate estimate of the PETCO_2_ change evoked by the BH task (Bright and Murphy, 2013). It must also be acknowledged that beginning and ending the BH period with exhalations may feel unnatural and be challenging to perform, as typically the participant will feel the need for deep inhalation following the BH. Special instructions and training (see below) are likely needed to achieve these important measurements.

Respiratory information and/or task compliance can also be assessed using a respiratory belt. However, studies comparing respiratory belt measurements with PETCO_2_ have shown that, although somewhat correlated, PETCO_2_ better models BOLD signal changes due to breathing tasks (Chang and Glover, 2009; Vogt et al., 2011).

#### Training

Pre-scan training is highly recommended when using task-based methods in order to ensure that the participant understands the instructions and how to perform the task (Magon et al., 2009; Kannurpatti et al., 2010; Zacà et al., 2014). Additionally, training also helps make the participant more familiar and less anxious about the task itself, improving compliance.

As part of the training, it may be desirable to explain what the participant should do if they feel unable to complete a trial. For example, if the individual needs to stop a BH prematurely, this can be readily tolerated, but it would improve data quality and interpretation to still perform a short exhalation at that time. This has the added benefit of giving participants more autonomy during the experiment, which may also reduce anxiety.

#### Task Instructions

Task instructions are usually provided using visual cues, although auditory cues can also be used (Riecker et al., 2003; Chang et al., 2008; Kannurpatti and Biswal, 2008; Tancredi and Hoge, 2013; Wu et al., 2019). Visual cues have ranged from simple written instructions displayed on a screen (Bright and Murphy, 2013; Haight et al., 2015; Lipp et al., 2015; Pinto et al., 2016; Liu et al., 2020), to color-coded or symbolic cues (Friedman et al., 2008; Thomason and Glover, 2008; Kannurpatti et al., 2010; Gonzales et al., 2014; Prilipko et al., 2014). In order to control for the potentially strong neuronal activation induced by visual instructions, brightness levels should remain similar across task and baseline periods (Kastrup et al., 1999a,b). An additional fMRI scan displaying the same instructions but without the execution of the breathing task can be performed in order to detect any differences in neuronal activation between task and baseline periods.

#### Data Analysis

Several strategies have been proposed to analyze fMRI responses to a vasoactive task, with focus on model-driven approaches, including the general linear model (GLM) approach. A box-car function describing the block design of the breathing paradigms may be used to build a model of the BOLD-fMRI response (Kastrup et al., 1999b; Biswal et al., 2007). Some studies have instead used ramp functions, assuming a linear increase in the BOLD signal with time in response to a BH task (Bright and Murphy, 2013). Rather than using the ideal/theoretical task paradigm, more precise models of the BOLD response can, in principle, be built based on the recorded respiratory traces, namely PETCO_2_ or respiratory belt data (Birn et al., 2008; Murphy et al., 2011; Bright and Murphy, 2013; Lipp et al., 2015). Using recorded respiratory traces to build the model has the advantage of intrinsically accounting for variations in task performance between subjects as well as within subjects across time. In particular, it has been demonstrated that the use of PETCO_2_-based models can account for irregular task performance, which is particularly relevant in non-compliant participants and thus has potentially great impact on patient studies (Bright and Murphy, 2013). The use of PETCO_2_ also becomes crucial for the normalization of CVR units (more details in the Other Considerations section).

In all cases, the temporal dynamics of the vascular response to the breathing task needs to be taken into account. This can be achieved by assuming a linear time-invariant system and convolving the task boxcar function, or the measured respiratory trace, with an appropriate impulse response function. Some studies have used the canonic hemodynamic response function (HRF) commonly used to describe the BOLD response to an impulse of neuronal activity; in this case, adding the temporal derivative to the model is critical to allow for the longer delays observed in the case of the respiratory response (Murphy et al., 2011; Jahanian et al., 2017). Birn and colleagues performed BOLD-fMRI measurements of single deep breaths and derived a new impulse response function to model the BOLD response to variations in the respiration volume per time (RVT), extracted from respiratory belt measurements – the Respiration Response Function (RRF). Using the RRF has been shown to provide a significantly better model of the BOLD changes induced by BH and PDB tasks than using the canonic HRF (Birn et al., 2008). In a later study, Vogt et al. derived an impulse response function of the BOLD signal to PETCO_2_ variations during paced hyperventilation (Vogt et al., 2011).

One aspect that most of these modeling strategies have in common is the assumption that a single, fixed time course can explain the BOLD signal across the brain. However, it has been suggested that the response to vasoactive stimuli may exhibit different temporal dynamics in different brain regions, not only in patients within cerebrovascular disease but also in healthy subjects (Chang et al., 2008; Magon et al., 2009; Geranmayeh et al., 2015; Pinto et al., 2016). Some studies have attempted to overcome this limitation by incorporating delay information into the analysis (Chang et al., 2008; Magon et al., 2009; Geranmayeh et al., 2015). The most common approach to obtain this information is by determining the optimal time lag within a certain time interval using cross-correlation between the voxel’s BOLD signal and the model (Chang et al., 2008). This optimization step can be performed voxelwise, or on a regional basis by considering a number of regions of interest (ROI) across the brain; this latter option may be advantageous in terms of SNR. Using the global BOLD timeseries as the reference has also been used to obtain an approximate estimate of the time delay of the BOLD response – but this does not take regional variations into account (Geranmayeh et al., 2015; van Niftrik et al., 2016). Other approaches that aim to estimate the voxelwise response time delay, include methods such as Hilbert transform (Raut et al., 2016), recursive tracking approaches such as the Regressor Interpolation at Progressive Time Delays method (RIPTiDe, RapidTide) (more details in the Resting-State Methods subsection) (Tong et al., 2011; Donahue et al., 2016; Champagne et al., 2019), iterative GLM fitting using shifted regressors (Geranmayeh et al., 2015; Cohen and Wang, 2019; Moia et al., 2020a), or Fourier basis modeling (Murphy et al., 2011; Lipp et al., 2015; Pinto et al., 2016; van Niftrik et al., 2016). The latter exploits the essentially biphasic shape of the BOLD response to alternating periods of task and baseline (assuming similar durations of each period), resulting in approximately sinusoidal signal variations at the paradigm frequency. More precise modeling of the shape of the response can be achieved by considering a Fourier series with a number of harmonics. Moreover, if a linear combination of a sine and cosine is used, then a phase delay can be estimated as well as the response amplitude. It has been reported that a sine-cosine pair at the task frequency and its two first harmonics provides a suitable model for the BOLD response to an end-inspiration BH task, yielding reproducible estimates of CVR amplitude as well as its time delay (Pinto et al., 2016).

The Fourier series approach has been shown to yield similar results to PETCO_2_-based modeling (Murphy et al., 2011; Lipp et al., 2015). This approach was further investigated by van Niftrik and colleagues in patients with unilateral hemispheric impaired perfusion (van Niftrik et al., 2016). Two sine waves with different frequencies were used in order to accommodate the fact that the BH and baseline periods had considerably different durations. Using the Fourier series approach to account for voxelwise optimal delay yielded significantly higher CVR values and better differentiation between affected and unaffected brain tissues (van Niftrik et al., 2016). Using iterative modeling of shifted regressors to account for voxelwise delay showed similar improvements in healthy controls (Moia et al., 2020a). Characterizing voxelwise delay information is clearly important for improving the accuracy of CVR measurements and may also supply complementary insight into the health or pathology of the local CVR response.

## Resting-State Methods

The last years have seen an increasing interest in the study of the brain’s intrinsic functional connectivity, based on synchronous fluctuations in the blood-oxygen level dependent (BOLD) signal across different brain regions (Biswal et al., 1995). The hemodynamically driven changes in tissue and blood oxygenation underlying the BOLD signal are, however, caused by a combination of neuronal activity as well as non-neuronal mechanisms, including respiration and cardiac sources. These fluctuations of non-neuronal origin are usually referred to as physiological noise in the context of conventional resting-state fMRI (rs-fMRI) studies of brain activity, and they are often modelled and eliminated as they are of no interest in those cases (Greicius et al., 2003; Birn, 2012; Pinto et al., 2017). However, this so-called physiological noise also contains information about cerebral hemodynamics that may be of interest, including CVR mechanisms.

In one approach to analyze rs-fMRI data, metrics are computed to quantify the amplitude of low-frequency oscillations, within the range of approximately 0.01 to 0.1 Hz (Biswal et al., 1995). Although spontaneous fluctuations in neuronal activity are thought to occur within this frequency range, non-neuronal physiological processes also contribute (Murphy et al., 2013; Bright and Murphy, 2015; Caballero-Gaudes and Reynolds, 2017; Tong et al., 2019a; Whittaker et al., 2019). In addition, it is well known that higher frequency fluctuations associated with the respiratory and cardiac cycles are aliased into the low-frequency range, due to the typically low temporal resolution of fMRI data, playing a significant role in spontaneous BOLD fluctuations. Furthermore, cardiac pulsations and the breathing cycle can lead to bulk motion of certain brain regions (Harvey et al., 2008) and spin history effects in the fMRI timeseries (Friston et al., 1996), while breathing-related chest movements can induce B_0_ field fluctuations that also influence the BOLD signal (Van de Moortele et al., 2002).

Some of the physiological fluctuations occurring within the low-frequency band are also thought to be governed by the autonomic nervous system. One source of such fluctuations, the variability of heart rate, is commonly used as a marker of autonomic nervous system activity (Chang et al., 2013). It has a higher frequency component thought to be related to parasympathetic modulation (0.15 - 0.4 Hz), and a lower frequency component (0.05 – 0.15 Hz) that appears to occur in synchrony with arterial blood pressure oscillations, also known as Mayer waves (∼ 0.1 Hz) (Julien, 2006; Draghici and Taylor, 2016). Another source of BOLD fluctuations in the low-frequency range is vasomotion caused by contraction of the smooth muscle of the arterioles, which is thought to be independent of cardiac and respiratory cycles (Murphy et al., 2013). Low- and very low-frequency BOLD fluctuations (0.023–0.73 Hz and 0.001–0.023 Hz, respectively) have also been associated with CSF pulsations (e.g., glymphatic system) (Mäkiranta et al., 2004; Kiviniemi et al., 2016). More recently, correlations between gastric oscillations (∼ 0.05 Hz) and low-frequency BOLD fluctuations have also been reported (Rebollo et al., 2018).

Pertinent to this review, natural fluctuations in PETCO_2_ levels also evoke low-frequency BOLD oscillations, providing more specific access to CVR. In 1997, Biswal et al. showed that the amplitude of low-frequency BOLD fluctuations was sensitive to hypercapnia (Biswal et al., 1997). In 2004, Wise et al. further demonstrated that low-frequency BOLD fluctuations across the human brain are temporally correlated with spontaneous PETCO_2_ fluctuations (Wise et al., 2004). Additional investigation confirmed that low-frequency BOLD fluctuations are also correlated with BH hypercapnic responses (Biswal et al., 2007; Kannurpatti and Biswal, 2008), in both younger and older populations (Kannurpatti et al., 2011).

Taking these physiological mechanisms into account, a new set of methods relying on the spontaneous variations of breathing and PETCO_2_ levels have been successfully used to assess CVR based on resting-state BOLD-fMRI. These methods have the advantage of not requiring any task to induce a vasoactive response and therefore being less demanding in terms of the experimental setup and more easily implemented, and also less dependent on the cooperation of the participant. This might be more suitable for CVR mapping in less cooperative populations, such as patients with language–comprehension problems or with visual/auditory impairment. Nevertheless, a limitation of this approach is that, if the subject’s spontaneous breathing pattern yields minimal fluctuations in their PETCO_2_ level, there might not be enough signal variation for reliable CVR assessment. In fact, de Vis and colleagues showed that a hypercapnic stimulus of at least 2 mmHg above baseline PETCO_2_ is necessary in order to evaluate any hemodynamic impairment (De Vis et al., 2018). Another challenge is isolating low-frequency BOLD fluctuations arising from CVR mechanisms versus other physiologic noise sources or neural processes described above.

Several metrics have been proposed to derive CVR information from rs-fMRI data, and they can be subdivided in two main categories: metrics based on s*ignal variation* or *signal regression*.

### Signal Variation

The resting-state fluctuation amplitude (RSFA) is a relatively simple metric commonly used in rs-fMRI studies, being computed as the temporal standard deviation of the BOLD time series (Kannurpatti and Biswal, 2008). Several studies comparing RSFA with CO_2_ inhalation and BH tasks for CVR estimation have reported similar results (Kannurpatti and Biswal, 2008; Kannurpatti et al., 2014; Wang et al., 2016), while other studies observed a low agreement between measures (Lipp et al., 2015). Nevertheless, RSFA has been shown to have high spatial repeatability (Lipp et al., 2015) and to be an important scaling factor in the analysis of the effects of age on task-fMRI (Tsvetanov et al., 2015).

Several other metrics based on modifications of the RSFA have been proposed as potentially more specific alternatives for CVR measurement. Makedonov et al. developed a physiological fluctuations (PF) metric, in which the temporal variance in signal attributed to thermal noise is subtracted from the total temporal variance of the BOLD signal at a particular voxel (Makedonov et al., 2013, 2016). Jahanian et al. proposed a coefficient of variation (CV) metric, in which the signal temporal standard deviation is normalized by the mean signal intensity (Jahanian et al., 2014, 2017). Jahanian et al. demonstrated that CV can be used to differentiate young healthy volunteers from hypertensive elderly subjects with chronic kidney disease. These CV differences mainly arose from very low-frequency components of the BOLD signal (< 0.025 Hz), and they became more significant after removal of physiological motion effects using the retrospective image-based correction (RETROICOR) method (Jahanian et al., 2014).

When comparing CV and BH-CVR, a strong linear correlation was observed in older adults (Jahanian et al., 2017). In contrast, moderate correlations were observed between CV and strategies based on PETCO_2_ signal regression (more details in the following subsection), although CV was best at representing whole-brain CVR variations across subjects (Golestani et al., 2016b). Liu et al. compared different physiological modulators of fMRI signal, obtaining a marginal correlation between CV and CVR using CO_2_ inhalation (Liu et al., 2013). The authors attributed this weak connection to CV being affected by factors such as bulk motion, spontaneous neural activity and breathing fluctuations (Liu et al., 2013).

Considering that some physiological fluctuations can be described within a well-defined frequency range, some authors have restricted their analysis to a specific low-frequency band. Usually, a frequency band of the 0.01 – 0.1 Hz or 0.01 – 0.08 Hz is chosen in order to target the components of interest while also removing high-frequency components possibly mostly contaminated by noise. The amplitude of low-frequency fluctuations (ALFF) metric is computed as the square root of the power spectrum across a specific low-frequency band of the preprocessed rs-fMRI BOLD signal (Zang et al., 2007). This metric has been used to rescale the amplitude of task-related fMRI in order to account for vascular differences (Kazan et al., 2016). More recently, the ALFF metric has been applied in patients with leukoaraiosis, yielding both decreases and increases in specific brain regions (Cheng et al., 2017). Another work on leukoaraiosis has shown that altered ALFF is positively correlated with executive function (Wang et al., 2019). Still, the low-frequency band is quite broad and more specific bands within this range may reflect different physiologic contributions. Zuo et al. (2010) demonstrated differences in the spatial distributions of ALFF in bands slow-2 (0.25–0.20 Hz), slow-3 (0.20–0.07 Hz), slow-4 (0.07–0.03 Hz) and slow-5 (0.03– 0.01 Hz). Although it remains challenging to discern what components of the low-frequency signals are specific to CVR mechanisms, correlation analysis with PETCO_2_ levels has been shown to be highest in the frequency band of 0.02–0.04 Hz (Liu et al., 2017a) (more details in the following subsection).

**FIGURE 4:**
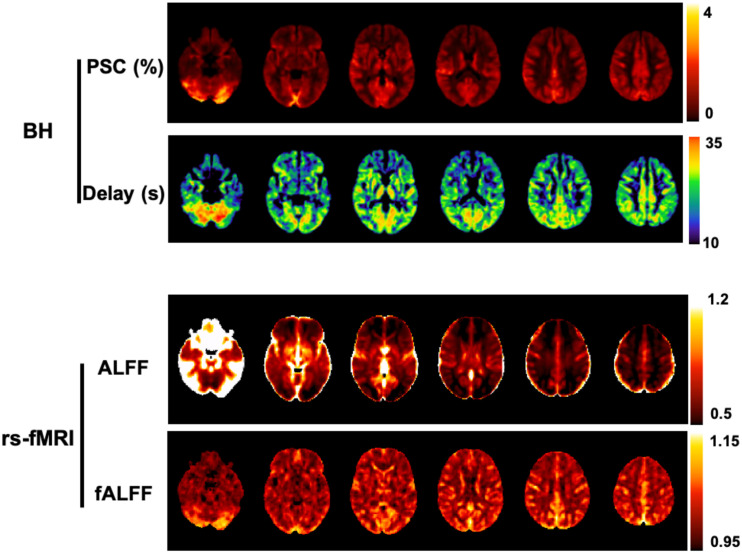
Comparison between BH and rs-fMRI protocols: **(Top)** CVR amplitude and delay maps obtained using BH BOLD-fMRI and the Fourier basis modeling approach (Pinto et al., 2016). **(Bottom)** Maps of ALFF and fALFF computed in the 0.01 - 0.023 Hz frequency band. All maps are group averages across 12 healthy individuals. For comparison purposes with the rs-fMRI metrics, the CVR amplitude maps are represented in non-normalized units of absolute BOLD percent signal change (PSC).

A related metric is the fractional ALFF (fALFF), whereby the power within the low-frequency range is normalized by the power across the whole spectrum (Zou et al., 2008). Both ALFF and fALFF have been shown to yield moderate to high test-retest reliability in gray matter (GM) regions, although ALFF is more reliable than fALFF (Zuo et al., 2010). Nevertheless, it has been demonstrated that ALFF is more prone to non-CVR physiological noise sources in comparison to fALFF, in particular near the ventricles and large blood vessels (Zou et al., 2008; Zuo et al., 2010). In summary, there is a tradeoff between reliability/specificity between the two metrics, and therefore some authors recommend reporting both ALFF and fALFF (Zuo et al., 2010; Figure 4).

Recently, De Vis and colleagues compared ALFF and fALFF metrics of rs-fMRI with CVR fMRI measurements during hypercapnia and hyperoxia gas challenges. A moderate relationship was found between hypercapnia and resting-state CVR measures in healthy individuals, but this relationship was decreased in a patient group. While hypercapnia fMRI measures led to large effect sizes when detecting hemodynamic impairment, rs-fMRI ALFF/fALFF metrics only showed moderate effect sizes. Nevertheless, in both hypercapnia and resting-state CVR measures, resting CBV (using hyperoxia BOLD signal as a proxy) significantly explained a large portion of BOLD signal variance across the brain (hypercapnia: 53/37%; ALFF: 31/23%; fALFF: 29/14%, in healthy controls/patients, respectively) (De Vis et al., 2018). Similar to ALFF, fALFF maps have also been employed as a voxelwise scaling factor of task-based fMRI analysis, significantly reducing inter-subject variability (Kalcher et al., 2013).

It should be noted that all signal variation metrics presented in this section are merely qualitative since they are not normalized against a common scale, usually the amplitude of PETCO_2_ fluctuations. Nevertheless, they can be sensitive to spatial differences since they are computed for each voxel separately. A summary of the most common rs-fMRI signal variation CVR metrics and their computation is depicted in Table 2.

**TABLE 2:** Summary of the most common rs-fMRI CVR methodologies based on BOLD signal variation metrics.

		Metric	Computation
Signal Variation	Whole spectrum	*RSFA*	σ_*EPI*_
		*CV*	$\frac{\sigma_{EPI}}{\mu_{EPI}}$
		*PF*	$\frac{\sqrt{\sigma_{EPI}^{2}-\sigma_{thermal}^{2}}}{\mu_{EPI}}$
	Low frequency	*ALFF*	$\frac{\sum_{k:f_{k}\left[f_{min};f_{max}\right]}\sqrt{\frac{{\left|FFT\right|}_{f_{k}}^{2}}{N}}}{n_{k}}$
		*fALFF*	$\frac{\text{ALFF}}{\begin{array}{c} \frac{\sum_{\text{m}:f_{m}\left[0_{};f_{max}\right]}\sqrt{\frac{{\left|FFT\right|}_{f_{m}}^{2}}{N}}}{n_{m}} \end{array}}$

*σ, temporal standard deviation; μ, temporal mean; $\sigma_{\mathrm{thermal}}^{2}$, temporal variance due to thermal noise; FFT, Fast Fourier transform; *f*, frequency range; *k*, number of bins within a specific frequency range; *m*, number of bins of the entire frequency range; *f*_*min*_, minimum frequency; *f*_*max*_, maximum frequency; *N*, number of time points; *n*, number of frequency bins.*

### Signal Regression

Given that PETCO_2_ is a well-established surrogate of arterial CO_2_, regression of BOLD signal by the spontaneous variations in this metric can also be used in order to estimate CVR in rs-fMRI (Wise et al., 2004). Another possible surrogate of arterial CO_2_ is the respiration volume per time unit, RVT (Birn et al., 2008; Chang and Glover, 2009). In 2009, Chang et al., 2009 verified that PETCO_2_ and RVT account for similar patterns of temporal and spatial variations of resting BOLD signal. However, the authors also observed differences between the two metrics, and indeed the BOLD signal variance explained was higher when using a combination of the two metrics compared to using only one of them (Chang and Glover, 2009). Several other studies have reported the use of PETCO_2_ as a regressor to map CVR based on rs-fMRI in healthy volunteers (Lipp et al., 2015; Golestani et al., 2016b) as well as in patients with cerebrovascular diseases (Liu et al., 2017a). Nevertheless, the rs-fMRI response to intrinsic PETCO_2_ fluctuations in healthy subjects may not be a reproducible metric, exhibiting lower intra-class correlation values than BH-CVR metrics (Lipp et al., 2015). In particular, the rs-fMRI response to PETCO_2_ fluctuations has been shown to vary with eyes-open and eyes-closed states (Peng et al., 2013). Golestani and colleagues investigated the impact of CVR mapping using PETCO_2_ in rs-fMRI after removing the cardiac and respiration contributions (Golestani et al., 2015, 2016b). They then estimated the relationship between rs-fMRI BOLD and PETCO_2_ fluctuations through parametric deconvolution, yielding a PETCO_2_-based response function (HRFCO_2_) (Golestani et al., 2015, 2016b).

In 2015, Liu et al. (2015) hypothesized that the average rs-fMRI BOLD signal across the whole brain reflects spontaneous arterial CO_2_ fluctuations, and employed global-signal regression (GSR) to derive CVR. This was validated in comparison with CO_2_-inhalation CVR maps. Furthermore, the authors also applied the proposed method to Moyamoya disease patients and found a reduced CVR in the diseased territories, with rs-fMRI CVR maps being comparable to those derived from CO_2_-inhalation (Liu et al., 2015). The same group also observed that rs-fMRI GSR methods provide significantly better CVR maps (in terms of Z-scores) compared to PETCO_2_ regression methods, and they speculate that this is due to the low sampling frequency of PETCO_2_ recordings (limited by breathing rate) compared to the BOLD signal (Liu et al., 2017a). More recently, this rs-fMRI GSR strategy was also applied in a group of stroke patients, confirming its feasibility (Taneja et al., 2019). Golestani and colleagues observed a good within-subject agreement between CVR values obtained using rs-fMRI GSR and conventional CO_2_ manipulation. However, rs-fMRI GSR was much less reproducible than methods based on rs-fMRI metrics, namely RSFA and ALFF. The authors state that one possible reason for this is the close relationship between GSR CVR estimates and frame-wise head motion (Golestani et al., 2016b). This confound may improve when isolating low-frequency components of the global signal. When different frequency bands (between 0 and 0.2 Hz) were taken into account, correlation between BOLD signal and PETCO_2_ was highest in the frequency band 0.02–0.04 Hz, yielding highly reproducible CVR maps that were spatially correlated with those obtained using the conventional CO_2_-CVR method (Liu et al., 2017a).

Regression using rs-fMRI averaged across a specific tissue mask has also been described by Jahanian et al. in a cohort of older adults (Jahanian et al., 2017), which may also facilitate the specificity of rs-fMRI signal regression to CVR mechanisms. In particular, when using the CSF signal, assuming it predominantly reflects fluctuations of purely non-neuronal origin, significant correlations with BH-derived CVR were observed, although these were lower than the CV method described earlier (Jahanian et al., 2017).

The temporal aspect of the low-frequency fluctuations of rs-fMRI has been also investigated with approaches primarily based on time-lag correlation, including simple seed-based lag mapping (Amemiya et al., 2014; Tong et al., 2017) as well as recursive tracking methods such as RIPTiDe (or its accelerated implementation, RapidTiDe) (Frederick et al., 2012; Erdoğan et al., 2016). The former generates a lag map based on the rs-fMRI time shift that yields the maximum correlation coefficients in each voxel relative to a predefined reference time course. In contrast, the latter approach takes into account the rs-fMRI data itself, with the reference time course being updated recursively, to better accommodate timing and shape differences. This is performed by shifting voxelwise the initial time course and applying principal component analysis or a weighted average to obtain an updated single time course that accounts for the highest shared variance. This approach might account for physiological differences in breathing responses and other possible factors that are difficult to know *a priori*. The use of optimized temporal delays at each voxel (using GSR) has been shown to increase the amount of BOLD signal variance explained relative to a GSR approach that does not perform this optimization (Erdoğan et al., 2016). Furthermore, the commonly seen negative correlations in GSR are minimized when using an optimized delay approach (Erdoğan et al., 2016). Although estimation of temporal delays may be less robust in rs-fMRI protocols compared to task-based CVR (Bright et al., 2017), rs-fMRI lag maps have revealed close correlation with SPECT-CVR maps, using acetazolamide as the vasoactive stimulus (Nishida et al., 2019). Some studies support the assumption that the delays of the low-frequency oscillations of rs-fMRI propagate in a way similar to cerebral blood circulation within the brain, reflecting vascular structure, both in healthy subjects (Tong et al., 2017, 2019b) as well as in a series of pathological conditions (Lv et al., 2013; Amemiya et al., 2014; Christen et al., 2015; Ni et al., 2017).

## Other Considerations

### PETCO_2_ as Surrogate of PaCO_2_

Estimation of PaCO_2_ during CVR assessment is a critical requirement for quantitative interpretation of results. However, direct measurement of PaCO_2_ requires blood sampling, making it highly invasive; for that reason, PETCO_2_ is generally used instead as a non-invasive surrogate. PETCO_2_ has been shown to provide a robust proxy of arterial CO_2_ partial pressure when using gas challenges that target PETCO_2_ levels in combination with respiratory rates greater than 12 breaths per minute (Ito et al., 2008). This assumption, however, might be compromised and not applicable in patients with diminished cardiopulmonary function, during exercise, or when using in combination with other breathing protocols (Peebles et al., 2007; Ito et al., 2008). Nevertheless, even if the absolute value of PETCO_2_ is not equivalent to the arterial CO_2_ content, its relative variation still accurately reflects the CO_2_ changes elicited by the stimulus (Mcswain et al., 2010). For these reasons, in practice, the use of PETCO_2_ as a surrogate of PaCO_2_ is a pragmatic choice for breathing modulation monitoring and analysis (Bright et al., 2019).

### Non-linear PETCO_2_ - CVR Relationship

The impact of baseline PETCO_2_ has been shown to influence CVR assessment (Halani et al., 2015; Golestani et al., 2016a; van Niftrik et al., 2018). In stroke patients, the compensatory baseline condition where vessels are dilated may explain the enhanced vasoconstrictive reactivity to hypocapnia in contrast to no significant changes in hypercapnic vasodilatory reactivity (Bright et al., 2011). Recently, it was demonstrated that CVR is significantly different when measured relative to a predefined “normocapnic” baseline (40 mmHg) compared to the subjects’ specific resting PETCO_2_ level (van Niftrik et al., 2018). Additionally, Hou and colleagues showed that subjects with higher baseline PETCO_2_ had lower CVR (Hou et al., 2020). Given all these observations, it is recommended that baseline PETCO_2_ levels should be reported in combination with CVR changes (Tancredi and Hoge, 2013; van Niftrik et al., 2018; Hou et al., 2020).

One possible explanation for why baseline PETCO_2_ level influences CVR measurement is the non-linear relationship between CBF (and the BOLD signal) and PETCO_2_. Although a linear relationship is usually assumed for CVR mapping, by applying progressive levels of hypo-/hypercapnia, a sigmoidal relationship provides a better model of the BOLD response (Bhogal et al., 2014; Sobczyk et al., 2014). Bhogal et al. observed a distinct BOLD signal plateau for high PETCO_2_ levels (assessed through gas challenges) (Bhogal et al., 2014), whereas Tancredi and colleagues emphasized that the CBF non-linear portion is critical mainly in the lower range of PETCO_2_ values (obtained by hyperventilation and ASL imaging) (Tancredi and Hoge, 2013). Combined, these observations indicate that baseline PETCO_2_ influences where on this non-linear, sigmoidal dose-response curve an individual is at rest and will thus influence the vascular response to any modulation in PETCO_2_ levels. It may be critically important to understand the role of non-linear CVR responses, particularly when comparing patient cohorts with different baseline physiology.

There is also substantial spatial heterogeneity of the non-linear BOLD response to PETCO_2_ (assessed through gas challenges) (Bhogal et al., 2014, 2015, 2016; Duffin et al., 2015; Fisher et al., 2017), emphasizing the need of studying CVR in a region-based or voxelwise manner. This heterogeneity is partly explained by differences in tissue types, anatomical regions and vascular territories (Bhogal et al., 2015).

### PETCO_2_ Normalization

Cerebrovascular reactivity can be expressed as percentage change in the fMRI signal normalized by the amplitude of concurrent PETCO_2_ variations. This normalization can minimize variability due to breathing task compliance differences across sessions and subjects (Bright and Murphy, 2013). When comparing BH-CVR with CO_2_-inhalation CVR, Kastrup and colleagues observed higher correlation when normalizing data with individual PETCO_2_ changes (Kastrup et al., 2001). However, Goode et al. found contradictory results when performing PETCO_2_ normalization on BOLD responses in a hypercapnic challenge, obtaining worse reproducibility and higher between-subject variability (Goode et al., 2009). Tancredi and colleagues also observed a reduction in CVR variability across different protocols (hypo- and hypercapnic, using ASL imaging) when CVR was expressed in terms of the percent change in CBF compared with normalized change. This was explained by the fact that normalized CVR values depend strongly on the range of PETCO_2_ levels considered, being greatly influenced by the non-linearity of the fMRI response to CO_2_ described earlier (Tancredi and Hoge, 2013).

In some cases, the PETCO_2_ normalization step is not mandatory and may not be useful; this is the case for example when using a representative ROI (e.g., lesion) time-series to compare with its contralateral location, in order to assess regional CVR alterations within the same individual. Additionally, CVR normalized in relation to whole brain or reference tissue values may be a more sensitive biomarker than absolute CVR in clinical applications, as it minimizes inter-subject variations (Yezhuvath et al., 2009). In general, methods based on resting-state signal variation are also not normalized against PETCO_2_ fluctuations. Still, when comparing CVR across individuals or cohorts, or tracking longitudinal changes in CVR, it is generally accepted that normalized CVR values are more appropriate (Liu et al., 2019).

### Role of O_2_

Within normal physiological values, the role of arterial O_2_ pressure in CBF regulation seems to be negligible (Ainslie and Duffin, 2009; Moreton et al., 2016), with reactivity to CO_2_ being approximately 60 times larger than to O_2_ (Prisman et al., 2008). Mark and colleagues observed no change in global CBF, as assessed by ASL, during an isocapnic hyperoxia stimulus (Mark et al., 2011). Breathing tasks, as described in this review, might also change arterial and venous O_2_ saturation levels and impact the BOLD signal (Bulte et al., 2009). Tancredi and colleagues also verified that, when using BH, the level of PETO_2_ decreased causing mild hypoxia; this could contribute a slight vasodilatory input, which would tend to exaggerate CVR measures. While the latter effect could explain the observed tendency of BH-CVR values to be higher than those for CO_2_ inspiration, the difference was fairly small and not statistically significant (Tancredi and Hoge, 2013).

### ASL

As previously described, the BOLD signal results from a complex combination of several physiological parameters, including CBF, CBV and blood oxygenation, although it is thought to reflect predominantly CBF changes. For direct, quantitative measures of CBF, the ASL technique can be used (Alsop et al., 2015; Pinto et al., 2020). Nevertheless, in CVR mapping, the main limitations of ASL are the intrinsically low signal-to-noise ratio (SNR) and poor temporal resolution, making this approach problematic when combined with short BH and PDB durations and associated non-steady-state vasoactive responses. Furthermore, the hypo- and hypercapnic states attained for CVR assessment might elicit changes in blood flow velocity, and hence labeling efficiency and bolus arrival time, as well as changes in arterial blood T_1_, and in this way compromise ASL perfusion quantification and CVR results (Aslan et al., 2010; Tancredi et al., 2012). To date, the number of studies employing ASL imaging in combination with breathing tasks for CVR evaluation is relatively low, particularly when compared to BOLD imaging (Kastrup et al., 1999b, 2001; Li et al., 1999a,b; Lu et al., 2003; MacIntosh et al., 2003; Leoni et al., 2012; Tancredi and Hoge, 2013; Prilipko et al., 2014; Haight et al., 2015; Cohen and Wang, 2019). Faster acquisition strategies with simultaneous ASL and BOLD imaging have shown promising results in CVR assessment based on breathing tasks (Cohen and Wang, 2019) and may increase the utility of ASL CVR mapping methods without gas inhalation in future research.

## Practical Guidelines

Despite the multitude of strategies and options that have been proposed and reported, as described in this review, the CVR literature is still insufficient to derive definitive conclusions regarding the best practices. Nevertheless, here we provide some practical guidelines based on available data on how to get started with CVR mapping without the use of gas challenges. We highlight the need for guidance depending on the type of population under study and equipment available as well as the expertise of the researchers.

**FIGURE 5:**
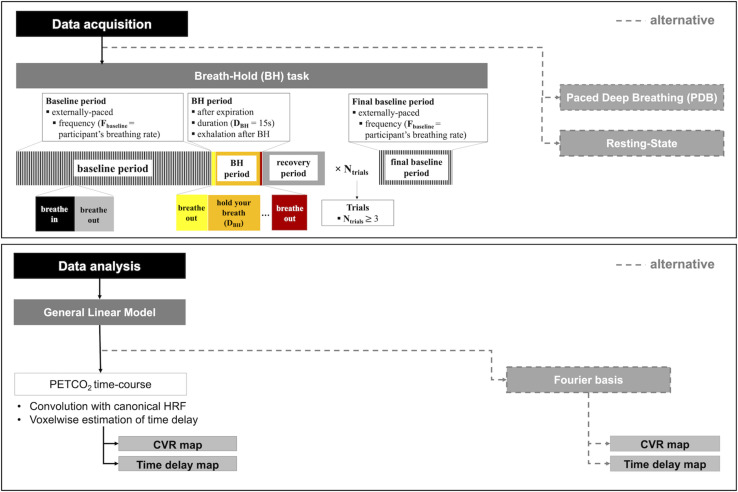
CVR mapping pipeline: recommended guidelines for data acquisition **(top)**, including the BH protocol with corresponding parameters, and data analysis **(bottom)**, including the GLM analysis, as well as recommended alternative approaches in each case.

First, the literature strongly indicates that a voluntary breathing manipulation (task-based) is able to produce larger CBF changes and hence more robust CVR measures than spontaneous breathing fluctuations (resting-state). To date, the most commonly used and most well studied breathing task is breath holding. For this reason, the choice of a BH task is recommended at this point, unless this is not feasible or otherwise appropriate for the population under study (see below). Several protocols have been proposed and tested, and we recommend the following parameters (illustrated in Figure 5): end-expiration BH; BH duration of 15s; exhalation immediately after the BH period to allow measuring PETCO_2_ at the end of the BH; recovery period with self-paced breathing; baseline period of externally paced breathing with a frequency that matches the participant’s breathing rate and a duration that allows blood gas levels to return to baseline; and a protocol with at least 3 BH trials.

Second, it is generally accepted that CVR metrics should be reported as a BOLD or CBF change normalized by the corresponding variation in PETCO_2_. We therefore recommend the recording of PETCO_2_ data throughout the MRI scan session: besides being crucial for this normalization, it also allows monitoring participant’s task compliance, assessment of baseline PETCO_2_ and estimation of both the PETCO_2_ sampling delay (if a brief BH followed by exhalation is performed) and regional hemodynamic delay. Furthermore, we also recommend the execution, prior to the MRI acquisition, of a practice run with PETCO_2_ recording. This will allow the assessment and optimization of the participant’s compliance as well as the retrieval of their natural breathing rate (to be incorporated in the BH protocol).

For data analysis, we recommend using the well-established GLM framework for fMRI. In fact, this is a relatively simple method and it can be easily implemented using widely available and finely tuned software tools (such as *FSL*^1^, *SPM*^2^, or *AFNI*^3^).

If PETCO_2_ data are collected, this should be pre-processed by end-tidal point extraction, interpolation, and sampling delay correction. For subsequent GLM analysis of the fMRI data, the resulting time course should then be convolved with a response function. Several studies have shown that the canonical HRF commonly used in fMRI studies is an appropriate choice and this can therefore be used for simplicity. Since variable temporal delays have been observed across the brain for the BOLD response to a BH, we recommend voxelwise estimation of this delay. This can be performed using the PETCO_2_ data, by applying the *RapidTide* method or the iterative shifted regressors GLM approach, for example.

If it is not possible to record the PETCO_2_, it should still be possible to obtain a non-normalized CVR map from a BH fMRI dataset. The GLM framework may again be used, but in this case we recommend using a Fourier-based description of the BH protocol, which depends only on the knowledge of the period of the task and not on a measured PETCO_2_ trace. In particular, we recommend using a GLM including a pair of sine and cosine regressors, together with their first two harmonics; this accounts for variable phases, or time delays, and has been shown to provide a good model of a BH BOLD response.

When studying pathologies where vessels are in a dilated state at baseline, hypercapnic/vasodilating challenges such as BH might not be appropriate. In these cases, PDB tasks eliciting a hypocapnic response may be more appropriate. Because the number of studies employing PDB tasks is still limited, it is not possible to make recommendations for the specific PDB protocol parameters based on the literature at this moment.

In the case of less cooperative participants, such as small children, and elderly or cognitively impaired patients, resting-state methods may be the best choice as they do not depend on task compliance. Nevertheless, again not enough data exist yet to allow the identification of one preferred acquisition and analysis protocol in this case.

In Figure 5 we summarize our recommended guidelines in a “CVR mapping pipeline”, that includes a BH protocol and GLM analysis, as well as the different alternatives.

## Future Work

Further investigation is still required in order to understand the physiological mechanisms underlying the BOLD response to changes in CO_2_ levels, as well as its spatiotemporal dynamics. In particular, it may be insightful to combine BOLD imaging with methodologies that measure neuronal activation (e.g., EEG) and/or CBF (e.g., ASL) directly, in order to allow the separation of different potential contributions to the measured BOLD response. Moreover, the more detailed and systematic investigation of the spatiotemporal dynamics of the BOLD response to CO_2_ variations might provide additional information of interest. The use of accelerated MRI strategies (e.g., simultaneous multi-slice acquisition schemes) providing higher temporal resolution might be advantageous for this purpose. Moreover, in resting-state studies, this may allow the separation of different physiological contributions to the BOLD signal by avoiding aliasing of faster fluctuations. Multi-echo acquisitions may offer further benefits, allowing for automatic decomposition of the data to isolate physiologic BOLD signal fluctuations from concurrent motion or other artifacts (Moia et al., 2020b).

In this work, we propose the use of a canonical HRF as the impulse response function when modeling the BOLD response to respiratory tasks. However, this aspect still requires further investigation: in analyses that do not include voxelwise optimization of vascular delay in the BOLD response, the latency of the response function would directly impact the resulting CVR amplitude estimates. While we recommend using an analysis method that accounts for differences in vascular transit and local response dynamics, it may still be beneficial to systematically explore the impact of different response functions, and potentially spatially-varying response functions, when analyzing these data (Golestani et al., 2015; Pinto et al., 2017; Kassinopoulos and Mitsis, 2019).

In terms of vasoactive modulation, more studies of PDB tasks are required to deliver sufficient data and allow more general conclusions. Combined breathing protocols should also be explored. In fact, a newly proposed breathing protocol based on rs-fMRI combined with intermittent breath modulation was indicated as another option for less cooperative participants (Liu et al., 2020). This strategy combines the large dynamic range of changes induced by breathing tasks with the easiness of resting-state methods. Another very recently presented approach implements breathing tasks at the beginning of the rs-fMRI acquisition to achieve similar benefits while maintaining a truly “resting-state” portion of the acquisition (Stickland et al., 2020).

Overall, the convergence of standardized practices remains crucial for the broader application of CVR methodologies without gas challenges. Future studies should therefore consider following the practical guidelines or alternative strategies outlined in this review as much as possible, and more studies should be conducted to assess the impact of protocol details on CVR mapping.

## Conclusion

In this work, we overviewed the methods used to map CVR using MRI based on task-induced and resting-state (spontaneous) breathing modulations. Validation studies are still required in order to derive definitive conclusions regarding the best practices. However, based on the methodological overview performed in this review, we outline a set of practical guidelines. In particular, we recommend the use of a BH task in combination with a GLM framework for data analysis, but other strategies can also be productively employed depending on the data available and population studied. Overall, we hope that this review will help motivate the wider and more consistent use of CVR mapping techniques without gas challenges.

## Author Contributions

JP and PF contributed to the conception and initial design of the manuscript. JP performed the search and selection of the relevant studies. JP wrote the manuscript. MB, DB, and PF contributed to the manuscript revision and editing. All authors read and approved the submitted version.

## Conflict of Interest

The authors declare that the research was conducted in the absence of any commercial or financial relationships that could be construed as a potential conflict of interest.

## Footnotes

1 https://fsl.fmrib.ox.ac.uk/fsl/fslwiki

2 https://www.fil.ion.ucl.ac.uk/spm/

3 https://afni.nimh.nih.gov/
